# Validity of activity monitors in health and chronic disease: a systematic review

**DOI:** 10.1186/1479-5868-9-84

**Published:** 2012-07-09

**Authors:** Hans Van Remoortel, Santiago Giavedoni, Yogini Raste, Chris Burtin, Zafeiris Louvaris, Elena Gimeno-Santos, Daniel Langer, Alastair Glendenning, Nicholas S Hopkinson, Ioannis Vogiatzis, Barry T Peterson, Frederick Wilson, Bridget Mann, Roberto Rabinovich, Milo A Puhan, Thierry Troosters

**Affiliations:** 1 Faculty of Kinesiology and Rehabilitation Sciences, Department of Rehabilitation Sciences, Katholieke Universiteit Leuven, Leuven, Belgium and Respiratory Division, UZ Gasthuisberg, Leuven, Belgium; 2 ELEGI Colt Laboratory, Centre for Inflammation Research, University of Edinburgh, Edinburgh, Scotland, United Kingdom; 3 NIHR Respiratory Biomedical Research Unit at Royal Brompton and Harefield NHS Foundation Trust and Imperial College, London, United Kingdom; 4 Department of Physical Education and Sports Sciences, Thorax Foundation, Research Centre of Intensive & Emergency Thoracic Medicine, Athens, Greece and National & Kapodistrian University of Athens, Athens, Greece; 5 Centre for Research in Environmental Epidemiology, Barcelona, Spain; 6 Global Health Economics and Outcomes Research, Novartis Horsham Research Centre, Horsham, United Kingdom; 7 Precision Medicine, Pfizer Worldwide Research and Development, Sandwich, Kent, United Kingdom; 8 Horten Centre for Patient-oriented Research, University Hospital of Zürich, Zürich, Switzerland; 9 Department of Epidemiology, Johns Hopkins Bloomberg School of Public Health, Baltimore, MD, USA; 10 PROactive consortium, Europe, Europe; 11 Respiratory Rehabilitation and Respiratory Division, UZ Gasthuisberg, Herestraat 49 bus 706, Onderwijs & Navorsing I, Labo Pneumologie, B-3000, Leuven, Belgium

**Keywords:** Chronic diseases, Doubly labelled water, Indirect calorimetry, Activity monitoring, Physical activity, Validation study, Systematic review

## Abstract

The assessment of physical activity in healthy populations and in those with chronic diseases is challenging. The aim of this systematic review was to identify whether available activity monitors (AM) have been appropriately validated for use in assessing physical activity in these groups. Following a systematic literature search we found 134 papers meeting the inclusion criteria; 40 conducted in a field setting (validation against doubly labelled water), 86 in a laboratory setting (validation against a metabolic cart, metabolic chamber) and 8 in a field and laboratory setting. Correlation coefficients between AM outcomes and energy expenditure (EE) by the criterion method (doubly labelled water and metabolic cart/chamber) and percentage mean differences between EE estimation from the monitor and EE measurement by the criterion method were extracted. Random-effects meta-analyses were performed to pool the results across studies where possible. Types of devices were compared using meta-regression analyses. Most validation studies had been performed in healthy adults (n = 118), with few carried out in patients with chronic diseases (n = 16). For total EE, correlation coefficients were statistically significantly lower in uniaxial compared to multisensor devices. For active EE, correlations were slightly but not significantly lower in uniaxial compared to triaxial and multisensor devices. Uniaxial devices tended to underestimate TEE (−12.07 (95%CI; -18.28 to −5.85) %) compared to triaxial (−6.85 (95%CI; -18.20 to 4.49) %, p = 0.37) and were statistically significantly less accurate than multisensor devices (−3.64 (95%CI; -8.97 to 1.70) %, p<0.001). TEE was underestimated during slow walking speeds in 69% of the lab validation studies compared to 37%, 30% and 37% of the studies during intermediate, fast walking speed and running, respectively. The high level of heterogeneity in the validation studies is only partly explained by the type of activity monitor and the activity monitor outcome. Triaxial and multisensor devices tend to be more valid monitors. Since activity monitors are less accurate at slow walking speeds and information about validated activity monitors in chronic disease populations is lacking, proper validation studies in these populations are needed prior to their inclusion in clinical trials.

## Systematic review

### Introduction

There is evidence that regular physical activity is associated with a reduced risk of mortality and contributes to the primary and secondary prevention of several chronic diseases [1]. For example, a reduced risk of coronary heart disease, cardiovascular disease, stroke and colon cancer has been reported in more active individuals [2]. In patients with chronic obstructive pulmonary disease (COPD), regular physical activity leads to a lower risk of both COPD related hospital admissions and mortality [3]. Physical activity limitation is a major problem in patients with chronic diseases and needs to be accurately measured if therapies aimed at improving this are to be properly evaluated. A range of devices are available for this purpose but most have been validated in young, healthy subjects and their applicability to older or unwell populations, where movements tend to be slower, is not well established.

Physical activity is defined as any bodily movement, produced by skeletal muscles, requiring energy expenditure [4]. Daily physical activity can be considered as “the totality of voluntary movement produced by skeletal muscles during everyday functioning” [5]. Estimates of daily physical activity can be obtained by different approaches; questionnaires, energy expenditure measurements and activity monitors. Questionnaires rely on the subject’s recollection of activities and allow categorization of patients by physical activity (very active, active, sedentary and inactive) [6], but may lack the precision needed to detect changes in physical activity on a day to day basis.

Daily physical activity can be expressed as an overall measure of active energy expenditure, using indirect calorimetry techniques such as doubly labelled water or metabolic carts. Although doubly labelled water is regarded as a criterion method, this technique does not quantify the duration, frequency and intensity of physical activity performed. Metabolic cart systems which measure expired O_2_ and CO_2_ however cannot be used over extended periods of time.

Physical activity can also be monitored directly using physical activity monitors. In general, three classes of activity monitors are being used increasingly in chronic disease populations (e.g. COPD): pedometers, accelerometers and integrated multisensor systems. Pedometers are devices which estimate the number of steps taken through mechanical or digital measurements in only the vertical plane. This is a limited measure of physical activity [7,8]. Accelerometers detect acceleration in one, two or three directions (uni-, bi- or triaxial accelerometers). These devices allow determination of the quantity and intensity of movements [9]. Integrated multisensor systems combine accelerometry with other sensors that capture body responses to exercise (e.g. heart rate or skin temperature) in an attempt to optimise physical activity assessments.

With the advancement of technology, the number of activity monitors available to measure physical activity is growing. However, despite these advances, it remains a challenge to assess physical activity in slowly moving patients (such as those with COPD, chronic heart failure and diabetes type II) [10-12]. In these patients small changes in physical activity are likely to be important effects of interventions aimed at enhancing physical activity. Therefore, in order for investigators to interpret the effect of interventions on physical activity, activity monitors that have been properly validated in these patient groups are needed.

In order to make evidence based statements on the validity of activity monitors, a systematic review was conducted to identify available activity monitors that have been validated in both healthy adults and chronic disease populations.

### Methods

#### Inclusion criteria

Studies meeting the following criteria were included: (1) Population: healthy adults and adults with a diagnosis of chronic disease in whom inactivity is a likely contributor to morbidity or a target for treatment, but whose locomotor function is relatively preserved (COPD, heart failure, diabetes type II, frail elderly, primary pulmonary hypertension, chronic low back pain, fibromyalgia syndrome, obesity). (2) Measurement: any commercially available activity monitor for outdoor activity monitoring from uniaxial to triaxial accelerometers and multisensor devices to tools incorporating spatial information (e.g. GPS) or other information on motion. (3) Study design: studies that evaluated the validity of an activity monitor, i.e. testing an activity monitor against a criterion method, such as indirect calorimetry. Two types of validation studies were included; field validation studies (validation of an activity monitor against doubly labelled water) and laboratory validation studies (validation of an activity monitor using a metabolic cart or metabolic chamber and/or manual step-counting or video observation). (4) Clinical trials using activity monitoring as an outcome and which might contain a reference to a validation paper were included for hand-searching. (5) A search window between 1^st^ of January 2000 until 1^st^ of March 2012 was selected in order to capture sensors in contemporary use. This approach still allowed for the identification of older validation studies (published before 2000) of devices in current use in clinical trials. Main exclusion criteria were 1) studies in children (subjects younger than 18 years), 2) studies in subjects with abnormal biomechanical movement patterns (e.g. cerebral palsy, lower limb amputation), 3) studies only investigating the number of steps using pedometers because of the inaccuracy in measurement of total energy expenditure [7] and lack of ability to measure physical activity patterns [8].

No language restrictions were used; any non-English studies retrieved through the literature search were translated to determine their appropriateness for inclusion.

#### Search strategy and systematic review

Eligible studies were identified by searching the following databases: MEDLINE, EMBASE and CINAHL. A librarian was consulted prior to initiating the search in order to identify appropriate search terms to describe the population (from healthy adults to patients with chronic disease), physical activity and activity monitoring. A combination of MeSH terms (MEDLINE), Emtree terms (Embase) and Cinahl headings (Cinahl) with free text words (all databases) were used (see Additional file 1 for detailed information). Refworks (www.refworks.com) was used to store and share all papers and to collect all the information of title and abstract screening, full text assessment and the hand-searching process. Each review team consisted of 3 reviewers who independently screened the titles and abstracts of the retrieved articles. Each abstract was labelled as ‘A) excluded papers‘, ‘B) order for full text assessment‘or ‘C) hand-search for references only’, i.e. clinical trials which may have a reference to an older validation study. After independently reviewing the articles for inclusion, the reviewers compared their labels to ensure consensus. Once agreement had been reached, a full text copy of each article that met the inclusion criteria was obtained (Label B). Thereafter, the same review teams looked at the full texts of the potential validation papers in detail and decided in consensus, whether the articles were indeed suitable validation papers for data extraction. Subsequently, hand-searching of the clinical trials using an activity monitor outcome which might contain a reference to a validation paper (Label C), was performed by three independent reviewers. After independently reviewing these full texts, validation papers were identified which met the inclusion criteria for full text assessment. Again, the reviewers compared their decisions to ensure consensus. Data of all included validation papers were extracted into predefined prepared Excel tables.

#### Data extraction

For the field studies, correlation coefficients between total and active energy expenditure from activity monitor (TEE_AM_ and AEE_AM_ respectively) and total and active energy expenditure measured with doubly labelled water (TEE_DLW_ and AEE_DLW_ respectively) were extracted. The percentage mean differences (ΔTEE and ΔAEE) with 95% confidence intervals were obtained from the reports to assess agreement between energy expenditure estimates from the activity monitor (TEE_AM_ and AEE_AM_) versus energy expenditure measures from doubly labelled water (TEE_DLW_ and AEE_DLW_). For the laboratory studies, correlation coefficients between activity monitor outcome and EE measured by metabolic cart/chamber were extracted. A sub-analysis included to compare correlation coefficients derived from walking based protocols to correlation coefficients derived from protocols based on activities of daily living. Agreement between energy expenditure outcomes from the activity monitor versus criterion method (indirect calorimetry) were extracted by the mean difference at different treadmill walking speeds; slow walk (<3.2 km/hr or 1 mph), intermediate speed walk (3.2-6.4 km/hr or 2–4 mph), fast walk (6.5-8.05 km/hr or 4–5 mph) and running (8.06-11 km/hr or 5–7 mph). Accuracy of steps measured by activity monitoring was expressed as the percentage mean difference between steps measured by an activity monitor versus actual steps measured by the criterion method (video observation and/or manual step counting).

#### Statistical analysis

Descriptive statistics were used to report information about type of activity monitor, activity monitor outcomes and studied population. Papers were separated by type of validation, ‘field validation papers’ (validation of an activity monitor against indirect calorimetry, using the doubly labelled water technique) and ‘lab validation papers’ (validation of an activity monitor against indirect calorimetry, using a metabolic cart, metabolic chamber or direct observation).

We also analysed the results separately per type of device (uni-, bi-, triaxial and multisensor devices). We performed (DerSimonian and Laird) random-effects meta-analyses to pool the correlation coefficients and mean differences across studies and expressed heterogeneity by the I^2^ statistic, which estimates the percentage of total variation between studies that is due to heterogeneity rather than chance. I^2^ is calculated from basic results obtained from a typical meta-analysis as I^2^ = 100% x (Q-df)/Q, where Q is Cochran’s heterogeneity statistic and df the degrees of freedom. Negative values of I^2^ are put equal to zero so that I^2^ lies between 0% en 100% with larger values showing larger heterogeneity. We used the Fisher r to z-transformation in order to pool normally distributed data (z scores) rather than the skewed distribution of Pearson correlation coefficients [13]. We back transformed the pooled z-scores to correlation coefficients for easier interpretation.

We used random-effects linear regression models (meta-regression analyses) with the studies’ results as the dependent variable (and considering each studies’ standard error) to compare the type of devices (covariate) and to assess the type of population (covariate) as a potential explanation for heterogeneity. For those few studies where no measures of variability were reported we imputed the median standard deviations of those studies where the standard deviation was available. We did not perform meta-analyses for the laboratory studies where none of the studies provided standard deviations for ΔTEE and ΔAEE but presented the point estimates as graphs. Coefficient of variation for TEE_DLW_ and AEE_DLW_ was calculated per study population to investigate whether the degree of variation in TEE_DLW_ and AEE_DLW_ affected the correlation coefficients and/or mean differences, (i.e. higher correlations/mean differences in populations with larger variation in TEE and/or AEE).

### Results

**Figure 1:**
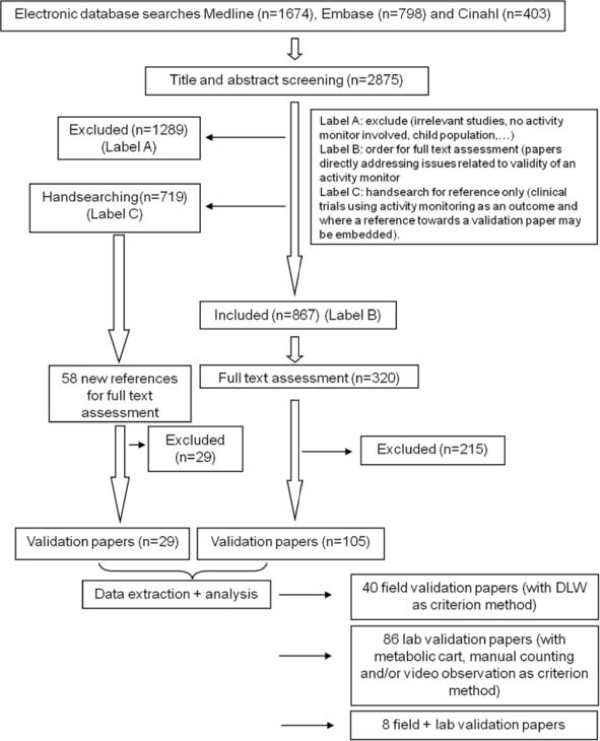
Flow chart describing the identification and inclusion of relevant studies.

The systematic literature search resulted in a total of 2875 abstracts which were scrutinised by four review teams across Europe. Figure 1 represents the different processes used in the systematic review.

Forty monitors were tested in validation studies; 12 uniaxial, 3 biaxial, 16 triaxial accelerometers and 9 multisensor devices. Fifty-five percent of activity monitors (22/40) were used only in lab validation studies, 10% (4/40) only in field validation studies and 35% (14/40) in both a lab as well as a field validation study. An overview of the different activity monitors is shown in Tables 1, 2, 3 and 4.

**Table 1:** Overview uniaxial activity monitors used in validation papers

**Name**	**Manufacturer**	**Field(F)/Lab(L) + reference**	**Size (and weight)**	**Placement**	**Cost**	**Epoch length**	**Data storage**	**Outcomes (measured)**	**Outcomes (calculated)**
**Actigraph Model 7164 (formerly CSA, MTI)**	Actigraph LLC Pensacola, FL	F [14-24] + L [7,25-47]	5.1 x 4.1 x 1.5 cm (45.5 g)	hip, ankle or wrist	NA	5 s to 1 min.	22 days (1 min epochs)	AC, steps	EE, activity intensity level
**Actigraph Model GT1M**	Actigraph LLC Pensacola, FL	F [48-50] + L [29,49,51-57]	3.8 x 3.7x 1.8 cm (27 g)	hip, ankle or wrist	€239 (unit), €249 (software)	1 s to several minutes	378 days (1 min epochs)	AC, steps	EE, activity intensity level
**Caltrac**	Muscle Dynamics Fitness Network, Torrance, USA	F [58-62] + L [7,34,35,63-65]	7 x 7 x 2 cm	waist	€71 (unit)	NA	no data storage	AC	EE
**Kenz Lifecorder EX**	Suzuken Co Ltd., Nagoya, Japan	F [66,67] + L [51,68-70]	7.25 x 4.15 x 2.75 cm (40 g)	waist	€49 (unit) + €250 (software)	5 s to 10 min	200 days	Steps, activity level	EE, activity intensity level
**Calorie Counter Select II**	Suzuken Co Ltd., Nagoya, Japan	L [7,71]	5 x 3 x 1 cm	waist	NA	1 day	7 days	steps	EE
**ActivPAL**	PAL Technologies Ltd, Glasgow, UK	L [57,72-74]	5 x 3,5 x 0,7 cm (15 g)	midline on the anterior aspect of the thigh	NA	1 s to 1 min	10 days	Steps (cadence), different body positions, activity score	
**PALlite**	PAL Technologies Ltd, Glasgow, UK	L [74]	5 x 3.5 x 0.7 cm (20 g)	ankle	€239	1 s to 1 min	10 days	Steps	
**PAM model AM101B.V.**	Doorwerth, Netherlands	L [37]	5.8 x 4.2 x 1.3 cm (28 g)	waist	NA	1 s to 1 min.	3 months	PAM scores	
**Actiwatch**	Mini Mitter Co, Sunriver, OR, USA	L [75]	4.4 x 2.3 x 1 cm (16.1 g)	wrist	€713 (unit), €213 (reader)	15 s to 1 min	30 days (1 min epochs)	AC	
**Biotrainer**	IM Systems, Baltimore, MD, USA	L [41,64,76]	7x 7 x 2 cm (51.1 g)	hip	NA	NA	9 days	AC	EE
**Nike and iPod sensor**	Apple Inc, Cupertino, CA, USA	L [77]	2,4 x 3,5 x 0,8 cm (9 g)	shoe	€19 (sensor)	NA	16 GB	Ground contact time	Distance, speed, EE
**Polar Activity Watch 200**	Polar Electro Oy, Kempele, Finland	L [78]	NA	wrist	€152 (watch + software)	1 min	Up to 9 files	Steps, HR	EE, activity intensity level

Field study (*F*), lab study ( *L*) or field + lab study ( *F + L*). *PAM*; physical activity monitor, *AC*; activity counts, *HR*; heart rate, *ECG*; electrocardiogram, *EE*; energy expenditure, *NA*; not available, *HR*; heart rate.

**Table 2:** Overview biaxial activity monitors used in validation papers

**Name**	**Manufacturer**	**Field(F)/Lab(L) + reference**	**Size (l x w x h) and (weight)**	**Placement**	**Cost**	**Epoch length**	**Data storage**	**Outcomes (measured)**	**Outcomes (calculated)**
**Biotrainer Pro**	IM Systems, Baltimore, MD, USA	L [32]	7.6 x 5 x 2.2 cm (51.1 g)	hip	€142 (unit), €142 (software), €70 (cable)	15 s to 5 min	22 days (1 min epochs)	AC, steps, activity intensity level	EE
**Actitrac**	IM Systems, Baltimore, MD, USA	L [76]	5.6 x 3.8 x 1.3 cm (34 g)	wrist	€570 (unit), €285 (software), €70 (cable)	2 s to 2 min	44 days (1 min epochs)	AC	
**AMP-331**	Activity Monitoring Pod, Dynastream Innovations Inc., Cochrane, AB, Canada	L [26,38]	7,13 x 2,4 x 3,75 cm (50 g)	right ankle (directly over the Achilles tendon)	NA	1 min epochs	28 hours (1 min epochs), 3.5 days (3 min epochs)	steps, cadence, walking speed, stride length, distance	EE

Field study (*F*), lab study ( *L*) or field + lab study ( *F + L*). *AC*; activity counts, *EE*; energy expenditure, *NA*; not available.

**Table 3:** Overview triaxial activity monitors used in validation papers

**Name**	**Manufacturer**	**Field(F)/Lab(L) + reference**	**Size (l x w x h) and (weight)**	**Placement**	**Cost**	**Epoch length**	**Data storage**	**Outcomes (measured)**	**Outcomes (calculated)**
**Actigraph GT3x**	Actigraph LLC Pensacola, FL	L [79]	4.6 x 3.3 x 1.5 cm (19 g)	Hp, ankle or wrist	€936 (device + software)	1 s to 1 min	19 days	VMU, steps	EE, activity intensity level
**RT3- Research Tracker**	Stayhealthy Inc. Monrovia, CA	F [80,81] + L [32,38,54,55,80,82,83]	7.1 x 5.6x 2.8 cm (65.2 g)	hip or waist	€142 per unit, €214 for docking station	1 s to 1 min	21 days	AC, VMU	EE
**TriTrac R3D**	Hemokinetics Inc, Madison, WI	F [14,16,80] + L [31-33,41,63,75,80,82,84-86]	10.8 x 6.8 x 3.3 cm (170.4 g)	waist	$500	1 min	14 days	AC, VMU	EE
**Tracmor**	Philips Research, Eindhoven, The Netherlands	F [87-94] + L [95-97]	7.2 x 2.6x 0.8 cm (22 g)	waist	€142 per unit, €214 for docking station	NA	21 days	AC	EE
**Tracmor**_**D**_**(Philips DirectLife)**	Philips New Wellness Solutions	F [98]	3,2 x 3,2 x 0,5 cm (12,5 g)	Lower back	€113	NA	22 weeks	AC	EE
**Dynaport activity monitor**	McRoberts BV, The Hague, The Netherlands	F [99]	12.5 x 9.5 x 3 cm (375 g)	waist + one leg sensor (thigh)	€4900 (+software)	1 s to 1 min	2 days (continuously) More days if SD memory card is used	movement intensity, different body positions	
**Dynaport minimod**	McRoberts BV, The Hague, The Netherlands	L [100,101]	8.5 x 5 x 1 cm (70 g)	waist	€1500 (unit)	1 s to 1 min	7 days	movement intensity, different body positions, steps	EE
**Biotel 3dNx**	Biotel Ltd, Bristol, UK	F [102] + L [29,103]	12.5 x 5.8 x 0.8 cm	hip or waist	€800	5 s to 60s	700 days	VMU	EE
**Actimarker, EW4800P**	Panasonic Electric Works Co Ltd, Osaka, Japan	F [67]	6 x 3.5 x 1.3 cm (24 g)	waist	€86 (device)	1 min	180 days	VMU	EE
**ActivTracer**	GMS, Tokyo, Japan	L [104]	4.8 x 6.7 x 1.6 cm (57 g)	waist				VMU	EE, activity intensity level
**Actical**	Mini Mitter Co, Sunriver, OR, USA	F [105] + L [26,38,44,54,106-112]	2.8 x 2.7 x 1.0 cm (17.5 g)	hip, ankle or wrist	€678 (incl. software)/€321 (unit)	15 s to 1 min.	45 days (1 min epochs)	AC, steps	EE, activity intensity level
**e-AR (earworn activity recognition sensor)**	Sensixa Ltd, London, UK	L [113]	5,6 x 3,5 x 1,0 cm (7.4 g)	ear	NA	1 min	NA	AC	EE
**PASE (Physical Activity Sensing Earpiece)**	MMA7260Q, Freescale Semiconductor, Austin, Texas	L [114]	0,6 x 0,6 x 0,14 cm (40 g, including data logging system)	ear	NA	15 s to 1 min.	NA	Acceleration units	EE, activity intensity level
**GENEA**	Unilever Discovery, Sharnbrook Bedfordshire, UK	F [115] + L [55]	3,6 x 3,0 x 1,2 cm (16 g)	Wrist, waist, ankle	NA	NA	8 days	VMU	EE
**Activity Style Pro HJA-350IT**	Omron Healthcare, Kyoto, Japan	L [116]	7.4 x 4.6 x 3.4 cm (60 g)	waist	NA	NA	NA	VMU	Activity intensity level
**CAM (Continuous Activity Monitor)**	Maastricht Instruments B.V.	L [117]	6.3 x 4.5 x 1.8 (102 g)	leg	NA	NA	NA	VMU	Activity intensity level, Different body positions

Field study (*F*), lab study ( *L*) or field + lab study ( *F + L*). *AC*; activity counts, *VMU*; vector magnitude units, *EE*; energy expenditure, *NA*; not available.

**Table 4:** Overview multisensor activity monitors used in validation papers

**Name**	**Manufacturer**	**Field(F)/Lab(L) + reference**	**Size (l x w x h) and (weight)**	**Placement**	**Cost**	**Epoch length**	**Data storage**	**Outcomes (measured)**	**Outcomes (calculated)**
**PAMS (Physical Activity Monitoring System)**	ICSensors 3031–010, Druck, The Netherlands	L [118]	5,0 x 3,0 x 0,8 cm (Tracmor, 16 g) + 4 tilt sensors (total weight = 1,3 kg)	lower back (Tracmor) + lateral aspect of the trunk and to the lateral aspect of the mid-thigh (sensors)	NA	NA	NA	voltage units	body position (lying, sitting, standing)
**Actireg**	Premed AS, Oslo, Norway	F [119,120] + L [46]	8.5 x 4.5 x 1.5 cm (60 g)	waist (storage unit) + chest and right thigh (sensors)	€440 (device) + €380 (software)	1 s to 1 min	30 days	body position and movement	Activity intensity level, EE
**Vitaport (+ 4 uniaxial accelerometers (ADXL202))**	University of Cologne, Cologne, Germany (Vitaport)/Analog devices, Breda, The Netherlands (Uniaxial accelerometers)	L [121]	1.5 x 1.5 x 1 cm (uniaxial accelerometer, 8 g)/6 x 11 x 3 cm (Vitaport, data recorder, 500 g)	4 sensors: 2 on skin of the ventral side of each thigh, 2 on the skin of the sternum,)	€ 15.000	1 s to 1 min	3 days	acceleration units	motility legs, motility trunk, motility body
**Sensewear Pro Armband (formerly Healthwear Armband)**	Bodymedia, Pittsburgh, PA, USA	F [50,122-126] + L [32,46,100,125-138]	8.8 x 5.6 x 2.1 cm (82 g)	Upper right arm at triceps (midhumerus point)	€800 (device) + €1597 (software)	1 min	14 days	Steps, activity intensity level	EE
**SenseWear Mini Armband**	Bodymedia, Pittsburgh, PA, USA	F [124]	NA	Upper left arm at triceps (midhumerus point)	€722 (device) + €1597 (software)	1 s to 1 min	28 days	Steps, activity intensity level	EE
**Actiheart**	Mini Mitter Sunriver, OR, USA	L [29,108,139]	0.5 x 1.1 x 2.2 cm (clip) + 10 cm (wire) (10 g)	3th intercostals space (clip) + 2 ECG electrodes (chest)	€1330	15 s to 1 min	11 days (1 min epochs)	Acceleration HR, HR variability, ECG amplitude	EE
**Ikcal**	Teltronic AG, Biberist, Switzerland	L [46]	NA	Chest (elastic belt around the sternum)	NA	NA	NA	Acceleration, HR	EE
**Multi-sensor board**	Department of Epidemiology, University of Washington, USA	L [111]	25 g	Hip	NA	1 s to 1 min	NA	Steps, activity intensity level, different body positions	EE
**IDEEA (Intelligent Device for Energy Expenditure and Activity)**	MiniSun, LLC, Fresno, CA, USA	F [140] + L [141]	7 x 5.4 x 1.7 cm (59 g) (recorder) + 1.8 x 1.5 x 0.3 cm (2 g) (sensor)	Waist (processing unit) + sole of both feet, both thighs and chest (sensors)	NA	1 s to 1 min	7 days	Activity code, speed, distance, power output	EE

Field study (*F*), lab study ( *L*) or field + lab study ( *F + L*). *AC*; activity counts, *EE*; energy expenditure, *NA*; not available.

The most frequently available outcomes present in validated activity monitors are (total and/or active) energy expenditure (70%, 28/40), steps (38%, 15/40) and different levels of physical activity intensity (38%, 15/40). The majority of the validation studies (118/134, 88%) were performed in healthy adults. Few studies (16/134, 12%) were performed in chronic disease populations; obesity (n = 4), chronic obstructive pulmonary disease (n = 5), chronic heart failure (n = 1), chronic organ failure (n = 1), chronic low back pain (n = 1), fibromyalgia syndrome (n = 1), peripheral arterial disease (n = 1), diabetes mellitus type II (n = 1) and a general chronic disease population (cardiac, obese or knee arthritis, n = 1).

#### Field validation studies

**Figure 2:**
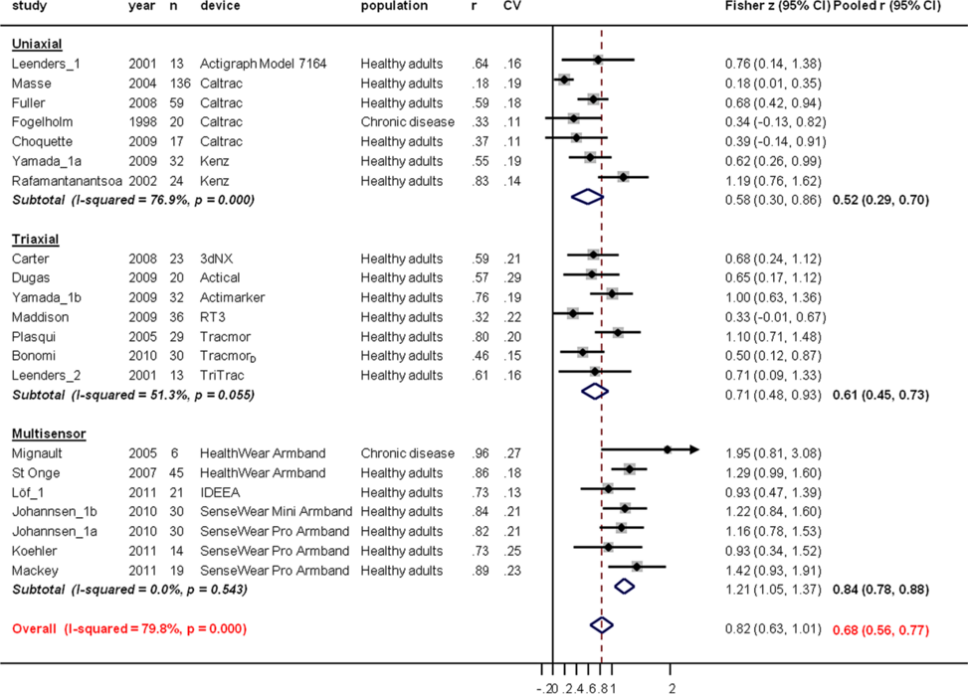
**Study-specific correlation coefficients (r) and Fisher z-scores (diamond) between total energy expenditure estimate from the activity monitor (TEE**_**AM**_**) and total energy expenditure measure from doubly labelled water (TEE**_**DLW**_**).** Each dot represents the z-score of the respective study together with a 95% confidence interval (CI) and the size of the box represents the weight of the study in the meta-analysis. Weights are from random effects analysis. CV; coefficient of variation for TEE_DLW_.

Individual correlation coefficients, with converted Fisher z-scores, for total energy expenditure (TEE) between TEE_AM_ and TEE_DLW_ are presented in Figure 2.

Variability of study populations’ TEE_DLW_ was relatively small; coefficient of variation (CV) ranged from 0.11 to 0.29. Pooled r in uniaxial devices (r = 0.52 (95%CI, 0.29 to 0.70)) was significantly lower compared to multisensor devices (r = 0.84 (95%CI, 0.78 to 0.88), p<0.001) but not to triaxial devices (r = 0.61 (95%CI, 0.45 to 0.73, p = 0.37)). Because of the relatively large difference in accuracy between the uniaxial, the triaxial and multisensor devices 53% of the between–study heterogeneity was accounted for by type of device in meta-regression analyses.

**Figure 3:**
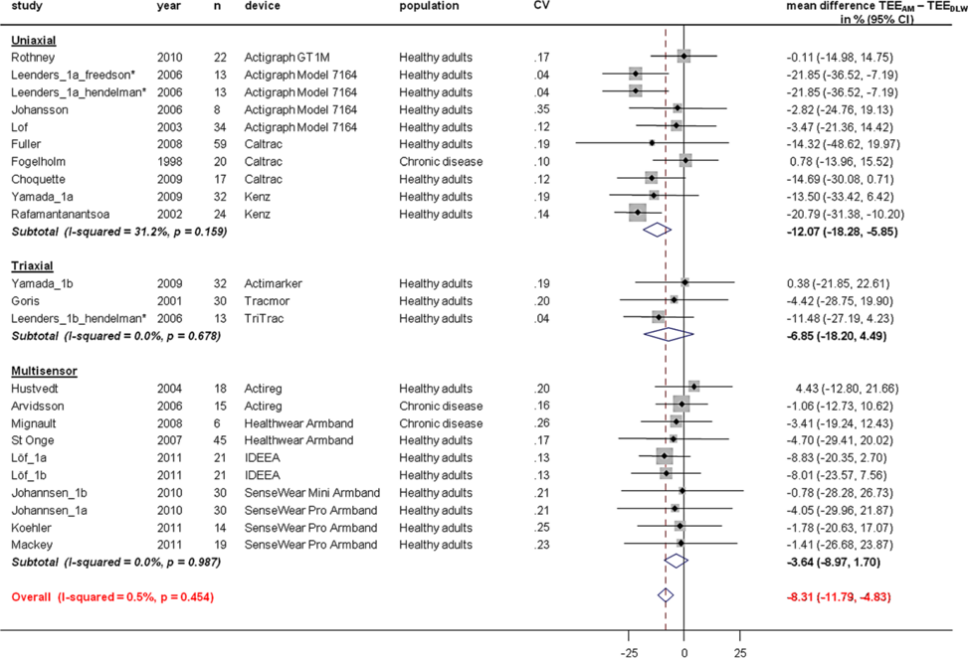
**Study-specific % mean difference (diamond) between total energy expenditure estimate from the activity monitor (TEE**_**AM**_**) and total energy expenditure measure from doubly labelled water (TEE**_**DLW**_**).** Each dot represents the mean difference of the respective study together with a 95% confidence interval (CI) and the size of the box represents the weight of the study in the meta-analysis. Weights are from random effects analysis. CV; coefficient of variation for TEE_DLW_. *Leenders et al. 2006 (Actigraph Model 7164); TEE_AM_ estimated with most frequently used Freedson and Hendelman equation (walking outdoors), (not reported) data of % mean difference (±SD) between TEE_AM_ - TEE_DLW_ with other previously published equations can be found in the original paper [14].

ΔTEE (TEE_AM_ – TEE_DLW_) was less accurate in uniaxial compared to triaxial accelerometers and multisensor devices (−12.07 (95%CI, -18.28 to −5.85) % in uniaxial versus −6.85 (95%CI, -18.20 to 4.49) % in triaxial (p = 0.39 for comparison against uniaxial devices) and −3.64 (95%CI, -8.97 to 1.70) % in multisensor devices, p = 0.03 for comparison against uniaxial devices, Figure 3). ΔTEE were smaller in studies with chronic disease populations than in studies with healthy populations (−9% (95%CI −19 to 1)) but the difference did not reach statistical significance (p = 0.09).

**Figure 4:**
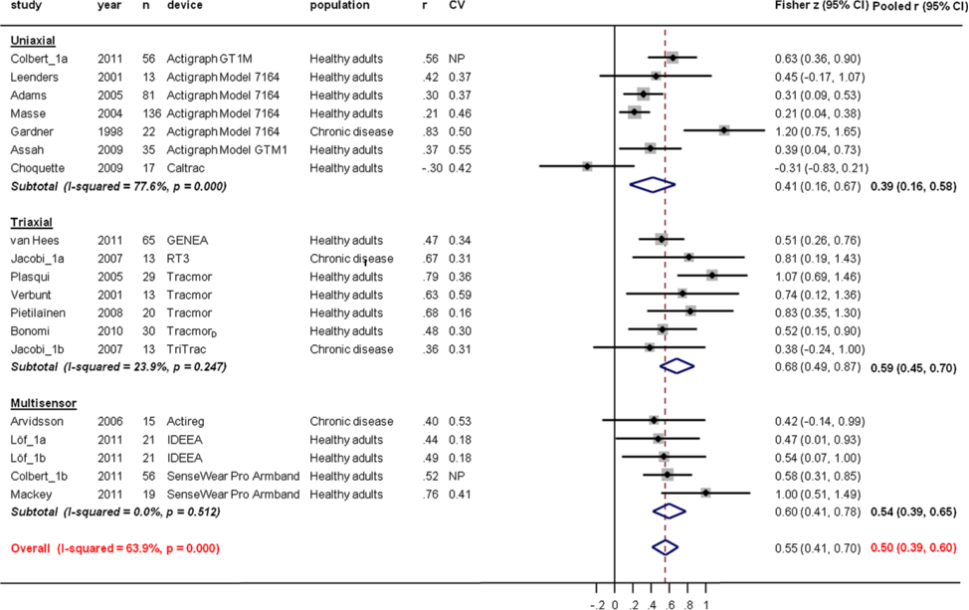
**Study-specific correlation coefficients and Fisher z-scores (diamond) between active energy expenditure estimate from the activity monitor (AEE**_**AM**_**) and active energy expenditure measure from doubly labelled water (AEE**_**DLW**_**).** Each dot represents the z-score of the respective study together with a 95% confidence interval (CI) and the size of the box represents the weight of the study in the meta-analysis. Weights are from random effects analysis. CV; coefficient of variation for AEE_DLW_.

**Figure 5:**
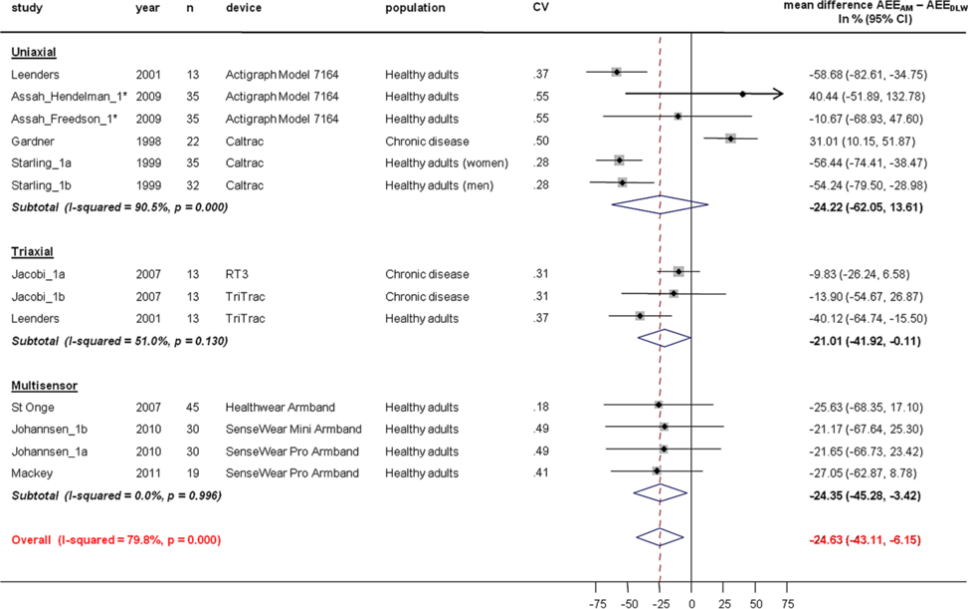
**Study-specific % mean difference (diamond) between active energy expenditure estimate from the activity monitor (AEE**_**AM**_**) and total energy expenditure measure from doubly labelled water (AEE**_**DLW**_**).** Each dot represents the mean difference of the respective study together with a 95% confidence interval (CI) and the size of the box represents the weight of the study in the meta-analysis. Weights are from random effects analysis. CV; coefficient of variation for AEE_DLW_. *Assah et al. 2009 (Actigraph Model 7164); AEE_AM_ estimated with most frequently used Freedson and Hendelman equation, (not reported) data of % mean difference between AEE_AM_ - AEE_DLW_ with other data derived and previously published equations can be found in the original paper [48].

Correlations for active energy expenditure (AEE) between AEE_AM_ and AEE_DLW_ were higher in triaxial (0.59 (95%CI, 0.45 to 0.70)) and multisensor devices (0.54 (95%CI, 0.39 to 0.65)) compared to uniaxial (0.39 (95%CI, 0.16 to 0.58)) devices, p = 0.12 for triaxial and p = 0.32 for multisensor against uniaxial devices) (Figure 4) Types of devices accounted for only 12% of the between-study heterogeneity in the meta-regression analysis. All monitors underestimated AEE (ΔAEE (AEE_AM_ – AEE_DLW_) -24.22 (95%CI, -62.05 to −13.61) % in uniaxial, -21.01 (95%CI, -41.92 to −0.11) % in triaxial and −24.35 (95%CI, -45.28 to −3.42) % in multisensor devices. No significant differences were found between devices (Figure 5). But ΔAEE were statistically significantly smaller in studies with chronic disease populations than in studies with healthy populations (−44%, 95%CI −73 to −13, p = 0.006).

#### Laboratory validation studies

For correlation analysis, TEE and AEE, as determined from indirect calorimetry, were used as criterion outcomes (in 89% and 11% of the studies, respectively) against different outcomes of the activity monitor (activity counts (37%), vector magnitude units (7%), total energy expenditure (48%), active energy expenditure (2%) or monitor-specific activity scores (6%).

**Figure 6:**
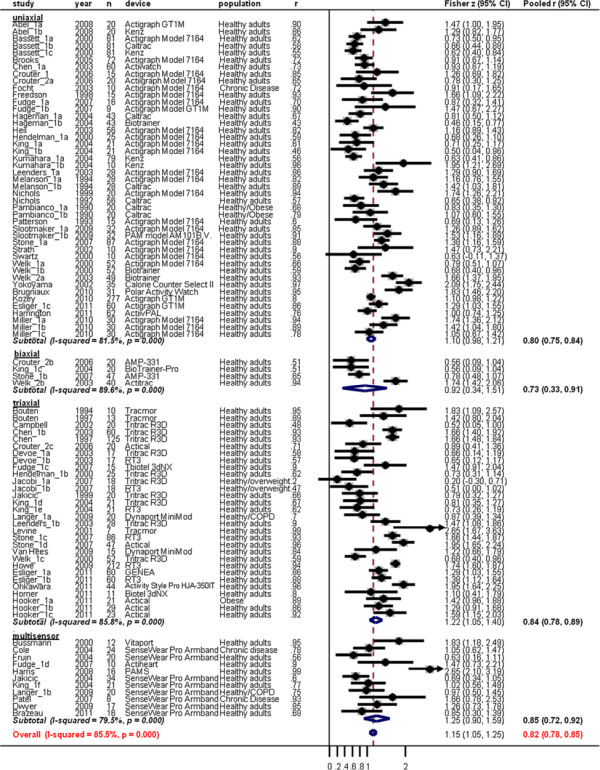
**Study-specific correlation coefficients (r) and Fisher z-scores (diamond) between activity monitor outcomes and total energy expenditure measure from indirect calorimetry (TEE**_**IC**_**) during laboratory protocols.** Each dot represents the z-score of the respective study together with a 95% confidence interval (CI) and the size of the box represents the weight of the study in the meta-analysis. Weights are from random effects analysis.

Pooled correlation coefficients between indirect calorimetry (TEE_IC_) and activity monitor outcome were lower in uniaxial (0.80 (95%CI, 0.75 to 0.84)) compared to multisensor devices (0.85 (95%CI, 0.72 to 0.92)) but the difference did not reach statistical significance (p = 0.43) No differences were found with biaxial (0.73 (95%CI, 0.33 to 0.91), p = 0.50) and triaxial (0.84 (95%CI, 0.78 to 0.89), p = 0.28) devices, either (Figure 6).

**Figure 7:**
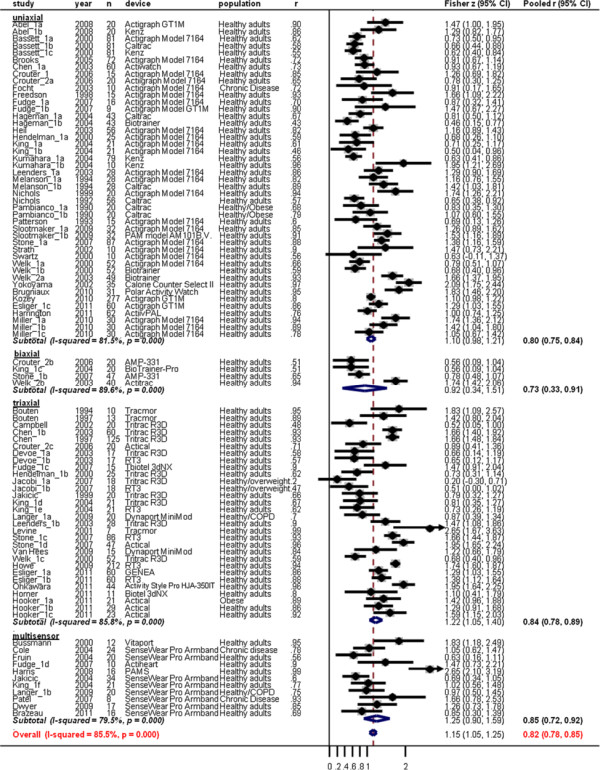
**Study-specific correlation coefficients and Fisher z-scores (diamond) between activity monitor outcomes and total energy expenditure measure from indirect calorimetry (TEE**_**IC**_**) during laboratory protocols based on walking activities.** Each dot represents the z-score of the respective study together with a 95% confidence interval (CI) and the size of the box represents the weight of the study in the meta-analysis. Weights are from random effects analysis.

**Figure 8:**
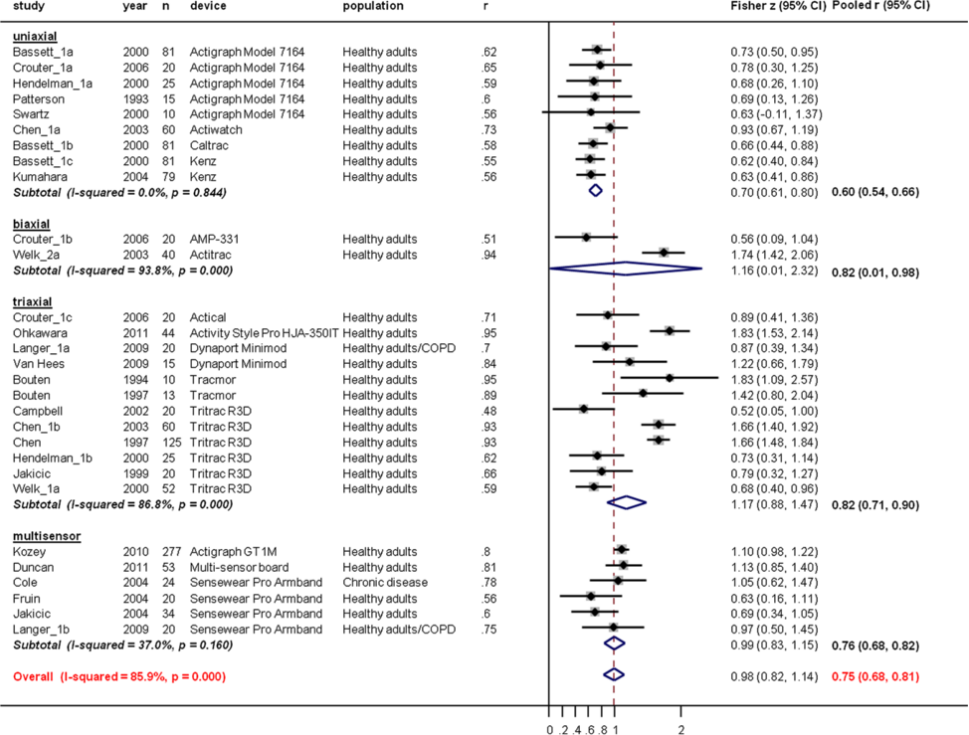
**Study-specific correlation coefficients and Fisher z-scores (diamond) between activity monitor outcomes and total energy expenditure measure from indirect calorimetry (TEE**_**IC**_**) during laboratory protocols based on activities of daily living activities involving the upper and lower limbs.** Each dot represents the z-score of the respective study together with a 95% confidence interval (CI) and the size of the box represents the weight of the study in the meta-analysis. Weights are from random effects analysis.

Correlation coefficients between TEE_IC_ and activity monitor outcome were higher when tested using laboratory protocols based on walking activities (overall pooled r = 0.84 (95%CI, 0.79 to 0.87), no significant differences between types of devices, Figure 7) compared to protocols using activities of daily living involving the upper and lower limbs (overall pooled r = 0.75 (95%CI, 0.68 to 0.81, no significant differences between types of devices), Figure 8).

There was evidence of heterogeneity of results across all analyses (overall I^2^ ranged from 84.6% (Figure 7) to 85.9% (Figure 8)). Again, the results did not differ for chronic disease and healthy populations in any of the analyses on laboratory validation studies.

**Figure 9:**
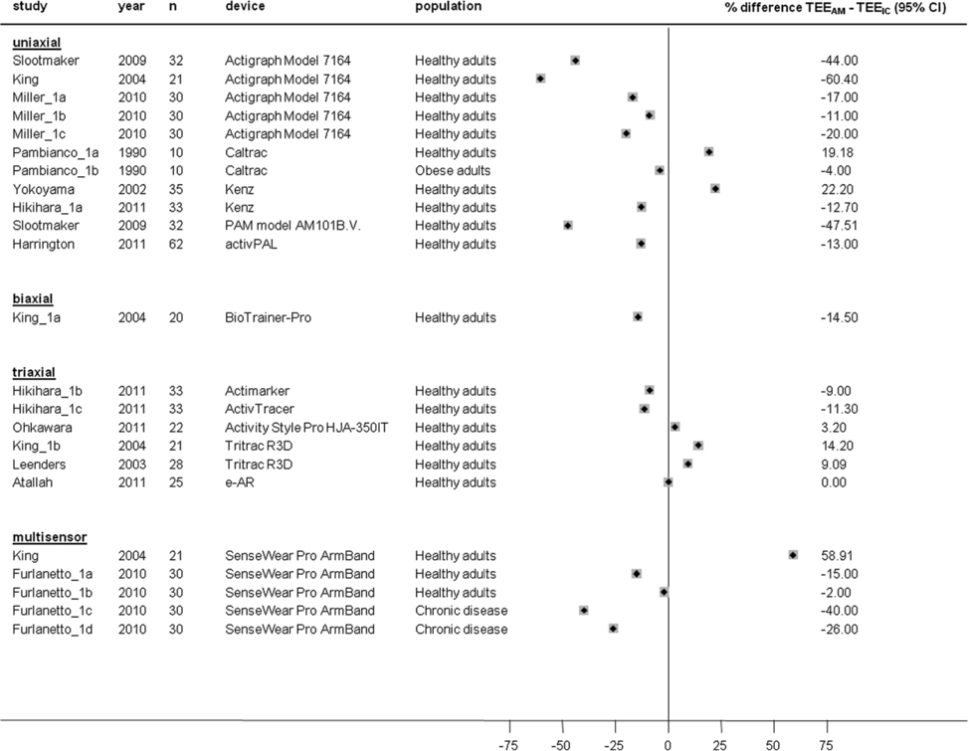
**Study-specific % mean difference (diamond) between total energy expenditure estimate from the activity monitor (TEE**_**AM**_**) and total energy expenditure measure from indirect calorimetry (TEE**_**IC**_**) during laboratory protocols based on slow walking speed.** Each dot represents the % mean difference of the respective study.

**Figure 10:**
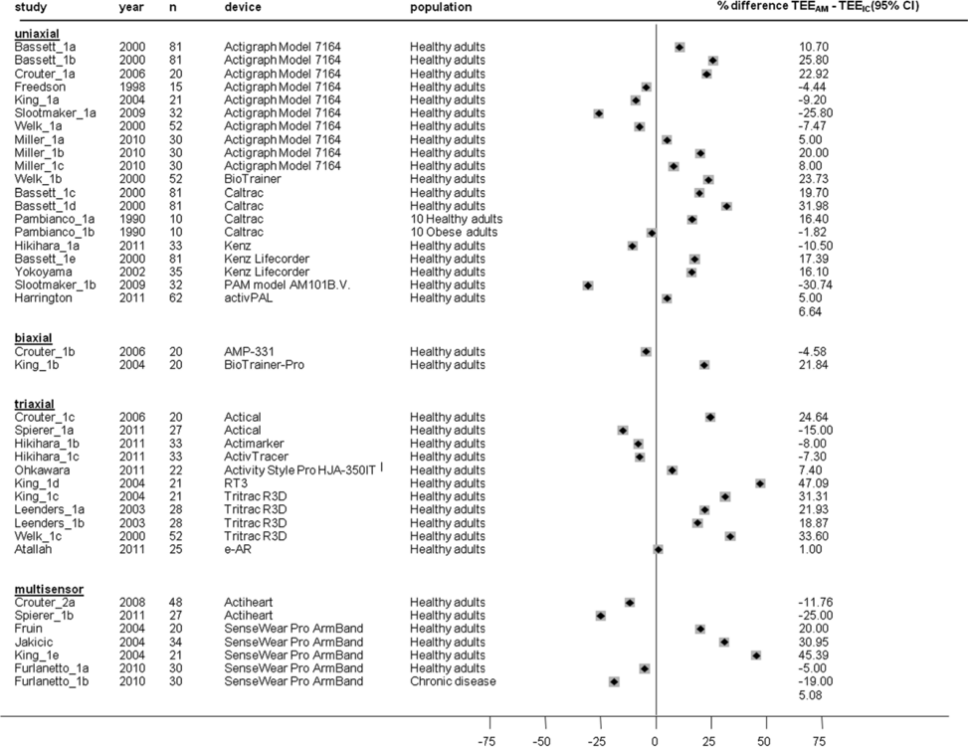
**Study-specific % mean difference (diamond) between total energy expenditure estimate from the activity monitor (TEE**_**AM**_**) and total energy expenditure measure from indirect calorimetry (TEE**_**IC**_**) during laboratory protocols based on intermediate walking speed.** Each dot represents the % mean difference of the respective study.

**Figure 11:**
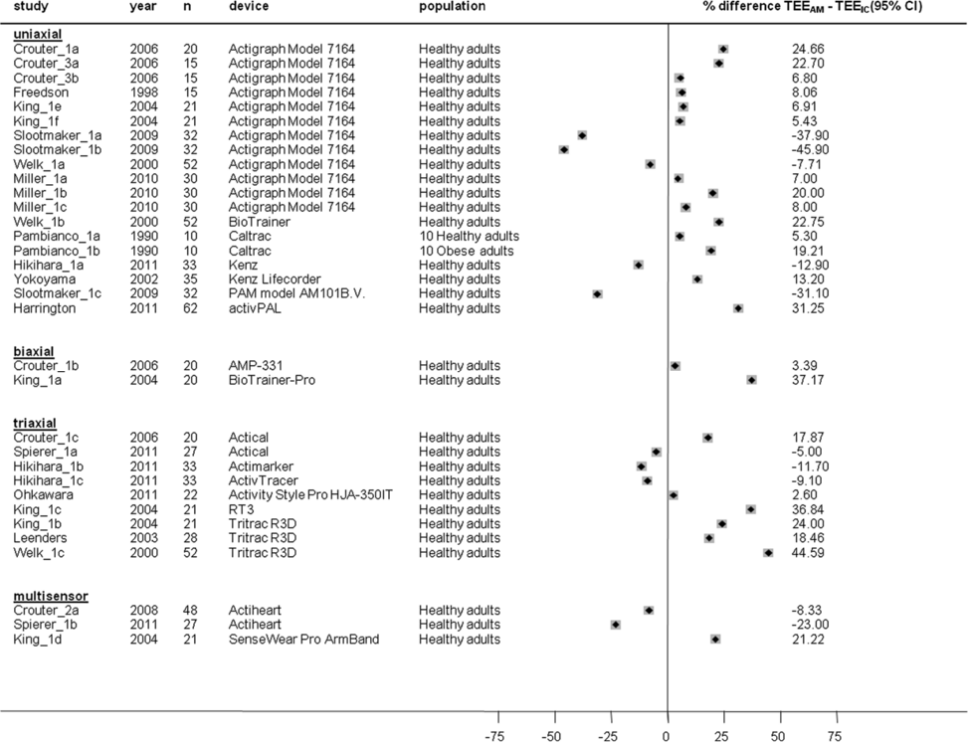
**Study-specific % mean difference (diamond) between total energy expenditure estimate from the activity monitor (TEE**_**AM**_**) and total energy expenditure measure from indirect calorimetry (TEE**_**IC**_**) during laboratory protocols based on fast walking speed.** Each dot represents the % mean difference of the respective study.

**Figure 12:**
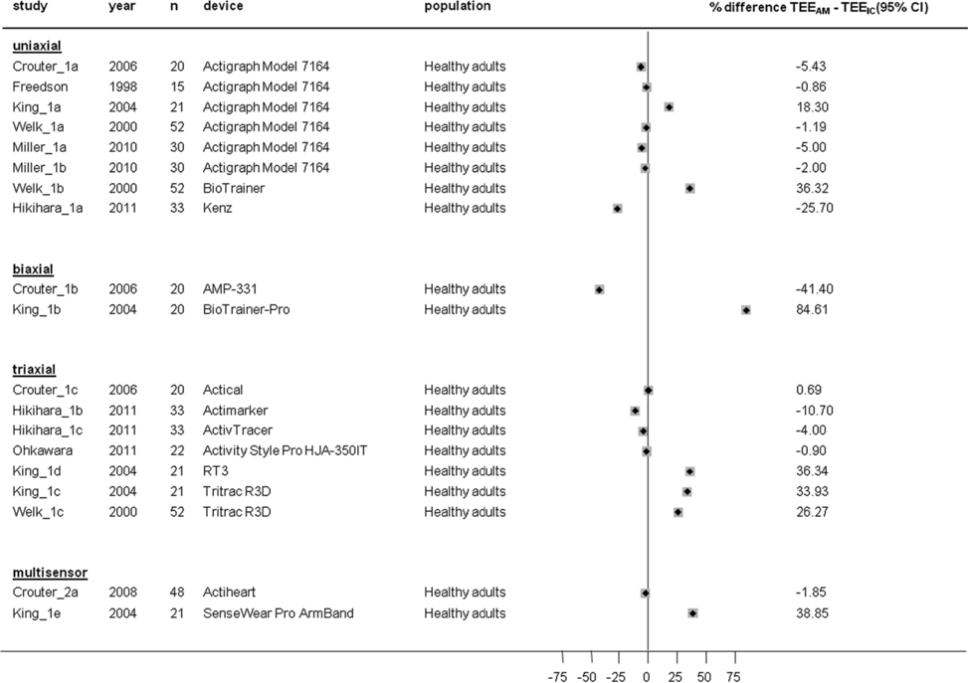
**Study-specific % mean difference (diamond) between total energy expenditure estimate from the activity monitor (TEE**_**AM**_**) and total energy expenditure measure from indirect calorimetry (TEE**_**IC**_**) during laboratory protocols based on running speed.** Each dot represents the % mean difference of the respective study.

Mean differences between TEE_AM_ and TEE_IC_ at different treadmill walking speeds are presented in Figures 9, 10, 11 and 12. TEE was underestimated during slow walking speed in 69% of studies (n = 16/23), whereas in only 37% of studies (n = 15/40) during intermediate walking speed, 30% of studies (n = 10/33) during fast walking speed and 37% of studies (n = 7/19) during running reported underestimation of TEE. Underestimations in the slow walking group were relatively larger.

**Figure 13:**
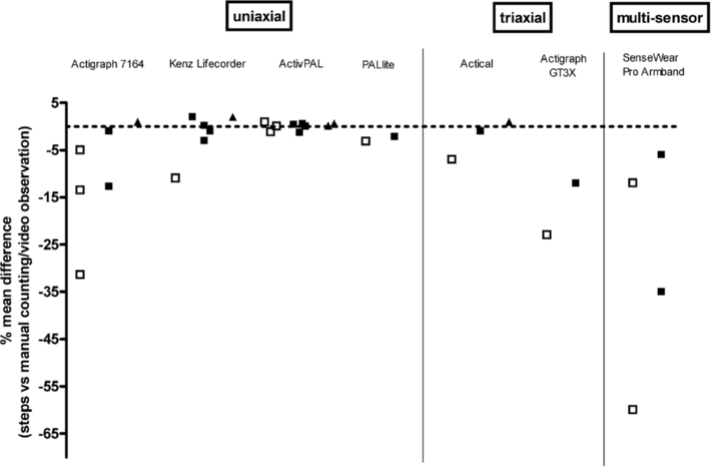
**Accuracy of steps at different walking speeds.** The dots are reflecting walking speed: slow walking (<3.2 km/hr (□)), intermediate walking (3.2-6.4 km/hr (■)) and fast walking (6.5-8 km/hr (▲)).

All accelerometers underestimate steps during slow walking; from 0.94 to 60% underestimation. One uniaxial device (activPAL), mounted on the thigh, showed a high accuracy in measuring steps during slow walking with only 0.94% overestimation. More accurate estimates of steps were reported at higher speeds; from 13% under to 2% overestimation during intermediate walking speed (except one study with 35% underestimation using SenseWear Armband), and from 0.18 to 4.3% overestimation during fast walking (Figure 13).

### Discussion

This systematic review of the literature identified forty activity monitors (12 uniaxial, 3 biaxial, 16 triaxial and 9 multisensor devices) that had been validated against indirect calorimetry (doubly labelled water, metabolic cart and/or metabolic chamber) in healthy adults (88% of studies) or adults with chronic disease (12% of studies).

Field and laboratory validation studies had highly heterogeneous results which could partly be explained by the type of activity monitor and the activity monitor outcome. These factors need consideration when a validation study is evaluated.

First, selecting the type of activity monitor is important. Pedometers are limited in their ability to detect certain physical activity patterns which might occur in chronic disease populations (for example, an unstable gait profile or lack of intensity of physical activity). Accelerometers can overcome this. Multi-axial accelerometers have the ability to measure accelerations in different orientations, which provides information about the total amount, intensity and duration of daily physical activity. Some multisensor devices, which combine physiological parameters with accelerometry, are available to assess both body posture and body movement. An additional promising class of monitors integrate positioning systems (Global Positioning System (GPS) and Bluetooth® systems for outdoor and indoor activities respectively) with accelerometry and other sensors. However, to date, these have been used infrequently in patients with chronic disease [142,143]. Based on this systematic review, heterogeneity among studies was significantly explained by the types of devices, although no statistical significance was reached between different types of devices.

A second factor to take into consideration is the activity monitor outcome. When measuring TEE in field validation studies (doubly labelled water), high correlations with the TEE estimate of the activity monitor were found in most activity monitors. These correlations are, however, to a large extent driven by patient characteristics (i.e. body weight, age, height) [87] which is an important predictor of TEE. Consequently, the comparison of TEE estimated from activity monitors, with TEE measured with indirect calorimetry or doubly labelled water is not necessarily a proof of validation. In a field setting it has been reported that only 19% of the TEE is accounted for by physical activity in both healthy subjects [87] and in patients with coronary heart disease [144].

Another factor that needs to be considered is the study population. Most of the study populations (88%) were healthy adults (from young healthy adults to healthy elderly). Only 12% of validation studies were performed in patients with chronic diseases (COPD, chronic heart failure, chronic organ failure, diabetes mellitus type II, obesity, peripheral arterial disease chronic low back pain and fibromyalgia syndrome). These patients walk more slowly than healthy subjects, which is reflected, for example, by a reduced six minute walking distance [145,146]. This review, as well as original research [147], suggests that most monitors are less accurate at lower walking speeds. These findings are consistent with a systematic review of pedometers which found evidence of reduced accuracy during slow walking [148]. Hence, there is a need to perform validation studies specifically in chronic disease populations.

When measuring TEE in lab validation studies by assessment of oxygen consumption, higher correlations were reported for walking activities compared to other daily life activities which implies that the walking component of physical activity is better detected than other activities of daily living.

Most activity monitors use prediction equations to calculate energy expenditure from the activity signals. This is helpful to validate monitors against indirect calorimetry, but, given the inherent inaccuracy of these estimates and fundamental differences between the different prediction equations (some of which are proprietary to particular device manufacturers), perhaps greater weight should be given to direct monitor outputs (steps, activity counts, VMU, etc.) and their relation to activity energy expenditure (AEE), rather than the ability of a monitor to estimate energy expenditure precisely [48,87-89]. It is very unlikely that an activity monitor will be able to capture accurately all the factors affecting energy expenditure (i.e. movement efficiency, resting metabolism, distribution of fat-free mass and fat mass). In patients with COPD, for example, Baarends et al. showed that non-resting energy expenditure (TEE-REE) was elevated in COPD compared to healthy controls [149]. Since it is generally accepted now that these patients are less active than healthy controls [150,151], it is clear that patients expend more energy than controls to achieve the same movements. It would be unrealistic to expect an activity monitor to pick this up. Hence, the lack of accuracy against energy expenditure does not render activity monitors invalid tools to assess physical activity in patients over time (for which precision is more important) or to capture the physical activity level of a patient (for which validity, represented by the correlation with true energy expenditure is more important than absolute accuracy). The acceptable correlations between VO_2_ and activity monitor outputs in triaxial and multisensor devices are therefore encouraging for the use of monitors to assess physical activity in an adult population. With specific validation studies, these findings can possibly be extrapolated to elderly and patients with chronic diseases.

The current systematic review may also help researchers to decide on appropriate activity monitor outcomes. Combination of the three most frequently available outcomes (TEE/AEE, steps and different levels of physical activity intensity), which is likely to provide a comprehensive insight in overall physical activity of a patient, is available in 3 uniaxial (Actigraph 7164/GT1M, Kenz Lifecorder EX and Polar Activity Watch 200), 1 biaxial (Biotrainer Pro), 3 triaxial (Dynaport Minimod, Actical and Actigraph GT3X) and 2 multisensor activity monitors (SenseWear Armband and multisensor board). Some general considerations can also be taken into account when selecting an activity monitor in clinical trials such as the type of monitoring (e.g. daily physical activity), size and scope of the study, usability of the monitor and cost [152].

#### Methodological issues

A point of difficulty in collecting, analysing and interpreting the data was the wide range of statistical approaches used in the original papers. Indeed, we had to compute the standard deviation of the mean difference (between EE_AM_ and EE_IC_) because some field validation studies didn’t report this.

Correlation analysis but also Bland and Altman analysis were the two main statistical approaches used in validation studies and were used for data extraction. A systematic review of the statistical methods used to validate physical activity questionnaires revealed similar findings, with the majority of the studies using correlation analysis compared to Bland and Altman analysis [153]. Correlation analyses are a common evaluation approach and allow statements on validity, whereas agreement between activity monitor and criterion method (indirect calorimetry) with Bland and Altman plots are preferred when the aim is to identify systematic bias in measures [154]. Since not all activity monitors have the possibility to estimate total and/or active energy expenditure, this type of analysis is not uniformly applicable. Multiple regression analysis with TEE/AEE as the dependent variable is a correct technique to tackle this [87]. Consistent statistical guidelines for reporting the validity of an activity monitor would be helpful.

### Conclusion

Validation studies of activity monitors are highly heterogeneous, and this is partly explained by the type of activity monitor and the activity monitor outcome. Since activity monitors are less accurate at slow walking speeds and information about validated activity monitors in chronic disease populations is lacking, proper validation studies in these populations are needed prior to their inclusion in clinical trials.

## Abbreviations

AEE, Active energy expenditure; AEEAM, Estimation of active energy expenditure by an activity monitor; AEEDLW, Measurement of active energy expenditure by doubly labelled water; AEEIC, Measurement of active energy expenditure by indirect calorimetry; AM, Activity monitor; COPD, Chronic obstructive pulmonary disease; DLW, Doubly labelled water; GPS, Global positioning system; IC, Indirect calorimetry; TEE, Total energy expenditure; TEEAM, Estimation of total energy expenditure by an activity monitor; TEEDLW, Measurement of total energy expenditure by doubly labelled water; TEEIC, Measurement of total energy expenditure by indirect calorimetry.

## Competing interests

The authors declare not having financial or non-financial competing interests.

## Authors’ contributions

All authors contributed to the different processes in the systematic review (title and abstract screening, full text assessment and data extraction). HVR worked out an appropriate search term strategy in different databases. HVR and SG have been involved in analysing the data and drafting the manuscript which was revised by all the other authors. MP performed the statistical analysis. All authors read and approved the final manuscript.

## Authors’ information

This systematic review is part of the PROactive project (www.proactivecopd.com) which aims to develop a Patient Reported Outcome (PRO) tool capturing physical activity in COPD in close harmony with a valid activity monitor.

## Supplementary Material

Additional file 1 Details of the search strategy terms in the different databases. Click here for file
