# Supplementary material for: Validity of activity monitors in health and chronic disease: a systematic review
Source: Int J Behav Nutr Phys Act. 2012 Jul 9;9:84. doi: 10.1186/1479-5868-9-84 (PMC3464146; doi:10.1186/1479-5868-9-84)
Supplement: Additional file 1 — Details of the search strategy terms in the different databases. [file 1479-5868-9-84-S1.doc]

**Methods**

**Details of the search strategy**

| **MEDLINE** |  |
| --- | --- |
| **Mesh terms (MEDLINE)** | **Free text terms** |
| heart failure | (heart OR cardiac OR myocardial OR congestive OR right-sided OR left-sided) AND failure |
| diabetes mellitus type 2 | ((non-insulin dependent OR type 2 OR slow-onset OR adult onset) AND diabetes mellitus)  OR (diabetes mellitus type II) OR (NIDDM) OR (diabetes mellitus type 2) |
| chronic disease | (chronic AND (disease OR illness)) OR (chronic AND (diseases OR illnesses)) |
| frail elderly | (frail AND elder* OR older adult)) OR (old* AND adult*) OR (old* AND people) OR (elderl*) OR adult* |
| chronic obstructive pulmonary disease | COPD OR Chronic Obstructive Pulmonary Disease OR Chronic Airflow Obstruction |
| pulmonary Hypertension | primary pulmonary hypertension |
|  |  |
| **AND** |  |
|  |  |
| exercise | (exercise* AND (physical OR aerobic)) OR (activity AND (physical OR aerobic OR daily living)) |
| activities of daily living | (activities AND (physical OR aerobic OR daily living)) OR ADL |
| bicycling | bicycl* |
| running | run* |
| walking | walk* |
| jogging | jog* |
| motor activity | (locomotion*) OR motor act* |
|  |  |
| **AND** |  |
|  |  |
| ambulatory Monitoring | (activity monitor*) OR (ambulatory monitor*) OR acceleromet* OR pedomet* OR step counter*  OR actigraph* OR sensewear armband OR caltrac OR dynaport OR minimod |
|  |  |
|  |  |
| **EMBASE** |  |
| **Emtree terms (EMBASE)** | **Free text terms** |
| heart failure | myocardial failure OR cardiac failure OR congestive failure OR congestive heart failure OR right sided failure OR  right sided heart failure OR left sided heart failure OR left sided failure |
| non insulin dependent diabetes mellitus | non insulin dependent diabetes mellitus OR type 2 diabetes mellitus OR slow onset diabetes mellitus OR  adult onset diabetes mellitus OR diabetes mellitus type II OR NIDDM |
| chronic disease | chronic diseases OR chronic illness OR chronic illnesses |
| frail elderly | frail elderly adult OR frail older adult* older adult* OR old* people OR adult* OR elderl* |
| chronic obstructive lung disease | copd OR chronic obstructive pulmonary disease OR coad OR chronic obstructive airway disease OR  chronic obstructive lung disease OR chronic airflow obstruction |
|  | primary pulmonary hypertension |
|  |  |
| **AND** |  |
|  |  |
| aerobic Exercise | physical exercise* OR aerobic exercise* OR physical activit* OR aerobic activit* |
| daily Life Activity |  |
| cycling | cycling |
| running | running |
| walking | walking |
| jogging | jogging |
|  |  |
| **AND** |  |
|  |  |
| ambulatory monitoring | activity monitor* OR ambulatory monitor* OR acceleromet* OR pedomet* OR step counter* OR actigraph OR  sensewear armband OR caltrac OR dynaport OR minimod |
|  |  |
|  |  |
| **CINAHL** |  |
| **Cinahl Headings** | **Free text terms** |
| heart failure, congestive+ | heart failure OR cardiac failure OR myocardial failure OR congestive failure OR right sided failure OR right sided heart failure OR  left sided failure OR left sided heart failure |
| diabetes mellitus, non-insulin dependent | non-insulin dependent diabetes OR type 2 diabetes OR slow onset diabetes OR adult onset diabetes OR  diabetes mellitus type II OR NIDDM |
|  | chronic disease* OR chronic illness* |
| lung diseases, obstructive OR bronchitis  OR emphysema | copd OR chronic obstructive pulmonary disease OR COAD OR chronic obstructive airway disease OR  chronic obstructive lung disease OR chronic airflow obstruction |
|  |  |
| frail elderly | frail elderl* OR frail older adult OR old* adult OR old* people OR elderl* OR adult* |
|  |  |
| **AND** |  |
|  |  |
| physical Activity | physical exercise* OR aerobic exercise* OR physicla activit* OR aerobic activit* |
| activities of Daily Living+ | activit* of daily living OR ADL |
| cycling | cycl* |
| walking | walk* |
| jogging | jog* |
| movement | locomotion* |
|  |  |
| **AND** |  |
|  |  |
| accelerometers | activity monitor* OR ambulatory monitor* OR acceleromet* OR step counter* OR actigraph* OR sensewear armband OR  caltrac OR dynaport OR minimod |
